# Effectiveness of Electroconvulsive Therapy for Preventing Relapse and Recurrence of Depression in Adults With Major Depressive Disorder: An Updated Meta-Analysis of Randomized Clinical Trials

**DOI:** 10.7759/cureus.35683

**Published:** 2023-03-02

**Authors:** Hassaan Dar, Kiranmayi Vuthaluru, Atunde Folajimi, Leladher Maheshwari, Jeet Shah, Mithum Senaratne, Guiomarly Pizzorno, Neelum Ali

**Affiliations:** 1 Research, Jinnah Medical and Dental College, Islamabad, PAK; 2 Medicine, Jawaharlal Nehru Medical College, Belgaum, IND; 3 Neurology, NES Healthcare, Aylesbury, GBR; 4 Medical School, Bahria University Medical and Dental College, Karachi, PAK; 5 Medicine and Surgery, Sir Sayajirao General Hospital, Ahmedabad, IND; 6 Medicine, University of Ruhuna, Matara, LKA; 7 General Medicine, University of Carabobo, Orlando, USA; 8 Internal Medicine, University of Health Sciences, Lahore, PAK

**Keywords:** meta-analysis, recurrence, relapse, depression, electroconvulsive therapy

## Abstract

The present meta-analysis aimed to assess the impact of electroconvulsive therapy (ECT) in preventing the relapse and recurrence of depression in adults with major depressive disorders. The study was conducted following the Preferred Reporting Items for Systematic Reviews and Meta-Analyses (PRISMA) guidelines. Two authors conducted a systematic search of online databases, such as PubMed, PsycINFO, and EMBASE, using keywords, such as “electroconvulsive therapy,” “depressive disorders,” and “recurrence.”

The primary outcome measure was the incidence of relapse and recurrence in adults with major depressive disorder who received ECT alone or in combination with an antidepressant medication compared to those who received antidepressant medication alone. The secondary outcome measure was the change in the Mini-Mental State Examination score from baseline to the end of the trial in both groups. A total of six articles were included in the meta-analysis.

The pooled rate of recurrence in the ECT group was 28.4% compared to 30.6% in the antidepressant group, with no significant difference between the two groups (risk ratio (RR) = 0.84, 95% confidence interval (CI) = 0.65-1.10, p = 0.21). However, subgroup analysis showed that the risk of recurrence was significantly lower in patients receiving ECT with antidepressant therapy compared to the antidepressant group alone (RR = 0.65, 95% CI = 0.45-0.93, p = 0.02). On the other hand, when ECT was given alone, the risk was higher in the ECT group compared to the antidepressant group; however, the difference was not statistically significant (RR = 1.17, 95% CI = 0.79-1.75).

In conclusion, the results of this meta-analysis suggest that ECT alone or in combination with an antidepressant medication does not significantly impact the incidence of recurrence in adults with major depressive disorder when compared to antidepressant medication alone.

## Introduction and background

Depressive disorders are associated with significant mortality and morbidity [1]. Depending on how they are diagnosed, a depressive episode is defined as a period of almost daily depressed mood, lack of energy or tiredness, and a loss of interest or enjoyment in activities. These symptoms are accompanied by other issues such as difficulty focusing, excessive guilt or feelings of worthlessness, hopelessness, recurrent thoughts of suicide, and changes in sleep or appetite [2]. The prevalence of depression varies between countries and regions and is estimated to average 14.6% among 10 developed countries and 11.1% among eight developing countries [3]. The first-line treatment for moderate-to-severe depression is usually antidepressants alone or in combination with psychotherapy. However, most individuals with depression do not achieve remission with initial treatment [4]. A clinical study found that 50% of patients had a recurrence within 1.5 years of recovery, with similar rates among those treated with antidepressants during the index depressive episode and those who were not [5]. Electroconvulsive therapy (ECT) is one of the most effective forms of treatment for inducing remission from depression [6].

ECT involves applying electricity to the scalp to induce a generalized tonic-clonic seizure [7]. Maintenance or continuation of ECT usually includes treatment provided once a week to once a month after the end of an acute ECT treatment [8], although it may also be considered after a non-ECT acute treatment course or occasionally less frequently [9]. After the acute treatment phase, ECT may be considered for the first six months, with maintenance ECT starting beyond that time. Modified ECT, which involves administering ECT while under anesthesia and taking a muscle relaxant, is considered best practice and reduces the risk of complications such as fractures, panic, and pain [9]. Non-randomized prospective studies, randomized controlled studies, retrospective studies, and case reports have shown that maintenance and continuous ECT are effective and well-tolerated in preventing recurrence and relapse [10].

ECT is recommended as a first-line treatment for acute depressive episodes with certain clinical features, such as catatonia and psychosis, or when a patient refuses to eat or drink [4]. Guidelines also support ECT as a first-line treatment for certain conditions, treatment-resistant depressive episodes, and in pregnant women, especially during the first trimester when medication is deemed contraindicated for treating depression [11].

With the understanding of the relapse and recurrence of depression after a successful course of acute ECT, many studies have been conducted over the last two decades. A systematic review supports the view that ECT plays an important role in mood disorder management [10], but some clinical trials have produced inconsistent results. Therefore, an updated meta-analysis of existing literature on this topic would provide a comprehensive understanding of the effectiveness of ECT in preventing relapse and recurrence in people with depression, inform clinical practice, and guide future research in this area. Thus, the current meta-analysis has been conducted to assess the impact of ECT in preventing relapse and recurrence of depression in adults with major depressive disorders.

## Review

Methodology

We performed this meta-analysis in accordance with Preferred Reporting Items for Systematic Reviews and Meta-Analyses (PRISMA) guidelines.

Search Strategy and Study Selection

Two authors conducted a systematic search using online databases such as PubMed, PsycINFO, and EMBASE to search for relevant articles. We searched the databases using the keywords “electroconvulsive therapy,” “depressive disorders,” and “recurrence.” No restrictions were placed on the year of publication. However, we limited our studies to articles that were published in the English language. All records were imported into the EndNote X9 library. After removing duplicates, abstract and title screening were performed by two authors independently. The full text of articles that passed the initial screening was retrieved and further reviewed for inclusion and exclusion criteria. Articles fulfilled the inclusion and exclusion criteria included in this meta-analysis. Any disagreement between the two authors during the study selection process was resolved through discussion.

Eligibility Criteria

We included studies conducted among individuals aged 18 years or older with unipolar and bipolar depression. We considered both unmodified ECT (ECT without anesthesia and modified ECT (ECT administered under general anesthesia). In addition, we included studies irrespective of whether ECT was given alone or in combination with the pharmaceutical or non-pharmaceutical intervention. We excluded observational studies, non-randomized trials, case reports, and case series. We excluded studies published in languages other than English.

The risk of bias in all randomized controlled trials (RCTs) was evaluated using Cochrane Handbook for Systematic Reviews of Interventions. We categorized each assessment of the risk of bias and data extraction potential source of bias as high, low, or unclear risk of bias. Risk of bias assessment was performed by two authors independently. Any disagreement between the two authors was resolved via discussion.

Data extraction was performed by one author using the predesigned data extraction form developed on Microsoft Excel. Data extracted included the first author’s name, study setting, year of publication, groups, sample size, follow-up duration, and patients’ characteristics. The second author cross-checked the data and entered it in RevMan for data analysis. Any discrepancy that occurred in this process was resolved via discussion.

Outcomes and Data Synthesis

The primary outcome measure in this meta-analysis was the incidence of recurrence and relapse in adults with major depressive disorder who received ECT alone or in combination with an antidepressant medication compared with those who received antidepressant medication alone. The secondary outcome measure was the change in the Mini-Mental State Examination (MMSE) score from baseline to the end of the trial in both groups. For data analysis, RevMan Version 5.4.1 (the Cochrane Collaboration, London, United Kingdom) was used for data analysis. The incidence of recurrence and relapse was compared using a risk ratio (RR) with a 95% confidence interval (95% CI) via a random-effects model, while the change in MMSE score was calculated using a mean difference (MD) with a 95% CI. The cut-off of the p-value was kept at 0.05. Heterogeneity was assessed using the I^2^ statistic and Cochran-Q statistics. A p-value <0.2 was considered significant for heterogeneity. Subgroup analysis was performed to compare the combination of ECT and antidepressant therapy with antidepressant therapy alone and ECT alone with antidepressant therapy.

Results

**Figure 1:**
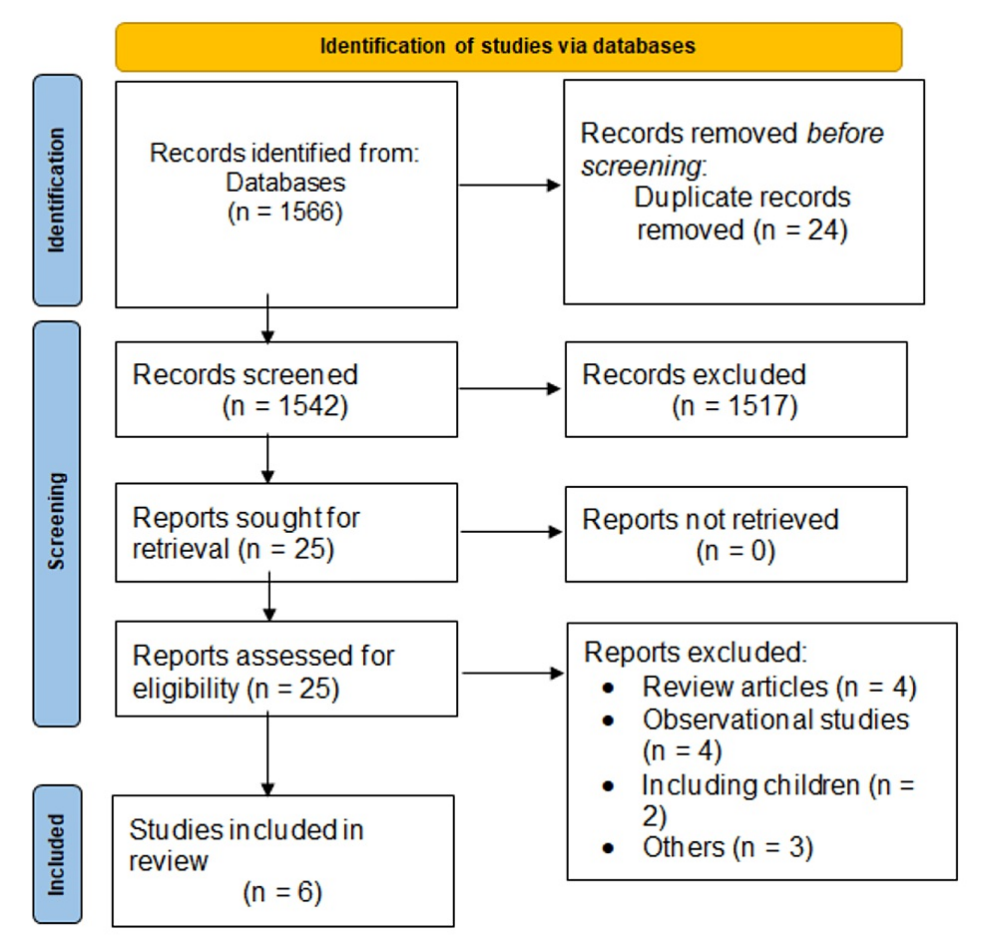
Preferred Reporting Items for Systematic Reviews and Meta-Analyses (PRISMA) flowchart of selection of studies.

The database search identified 1,566 records. After removing duplicates, the initial screening of titles and abstracts of 1,542 articles was done using the predefined inclusion and exclusion criteria. Full texts of 25 articles were retrieved and a detailed evaluation was done to assess their eligibility criteria. In total, six articles were included in this meta-analysis [12-17]. Figure 1 shows the process of study selection.

**Figure 2:**
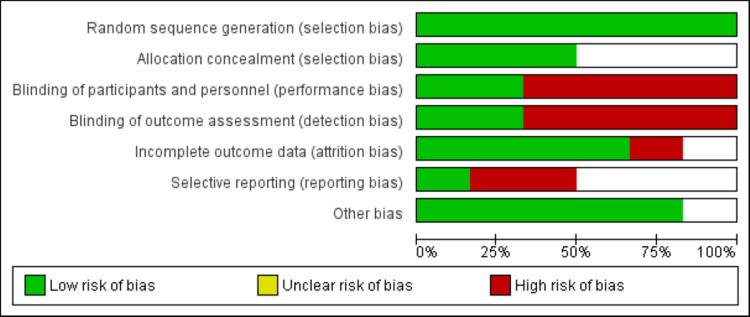
Risk of bias assessment.

The characteristics of included studies are shown in Table 1. One study investigated ECT as a monotherapy for recurrence or relapse prevention [14] and five studies examined ECT in combination with antidepressant drugs [12-17]. Five studies used the clinician-administered Hamilton Rating Scale for Depression (HRSD) in the 17, 21, or 24-item form along with other signs of worsening of the condition [12-16], while one RCT used the Montgomery-Asberg Depression Rating Scale (MADRS) [17]. Five RCTs included patients with unipolar depression [12-16], while one RCT enrolled unipolar as well as bipolar depressive disorder patients [17]. Figure 2 shows the risk of assessment of bias summary graph.

**Table 1:** Characteristics of included studies. ECT = electroconvulsive therapy; HRSD = Hamilton Rating Scale for Depression; MADRS = Montgomery-Asberg Depression Rating Scale

Author name	Year	Depression type	Groups	Co-intervention	Sample size	Follow-up	Mean age (years)	Males	Scale used for recurrence
Brakemeier et al [12]	2013	Unipolar depression	ECT	Pharmacotherapy	25	12 Months	60.2	32.6%	HRSD-24
			Pharmacotherapy		18				
Kellner et al [13]	2016	Unipolar depression	ECT	Pharmacotherapy	61	6 Months	70.5	38.3%	HRSD-24
			Pharmacotherapy		59				
Kellner et al [14]	2006	Unipolar depression	ECT	NA	89	7 Months	57.2	32.1%	HRSD-24
			Pharmacotherapy		95				
Martínez-Amorós et al [15]	2021	Unipolar depression	ECT	Pharmacotherapy	17	9 Months	68	35.3%	HRSD-21
			Pharmacotherapy		17				
Navarro et al [16]	2008	Unipolar depression	ECT	Pharmacotherapy	16	24 Months	70.5	51.5%	HRSD-17
			Pharmacotherapy		17				
Nordenskjöld et al [17]	2013	Unipolar and bipolar depression	ECT	Pharmacotherapy	28	12 Months	57	50.0%	MADRS
			Pharmacotherapy		28				

**Figure 3:**
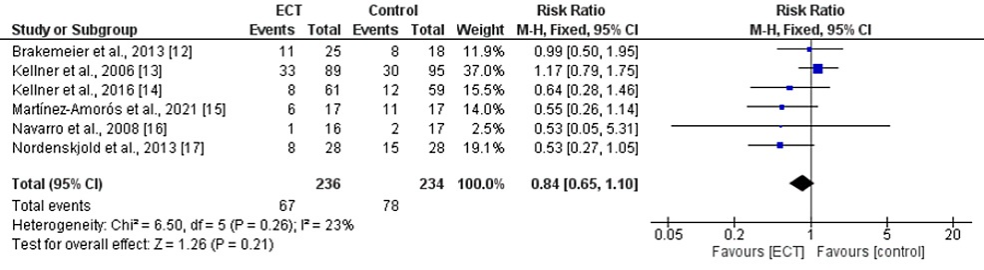
Forest plot showing the effect of ECT on recurrence and remission. ECT = electroconvulsive therapy Sources: references [12-17].

Six RCTs studied the effectiveness of ECT in combination with an antidepressant medication compared to an antidepressant alone. The pooled rate of recurrence in the ECT group was 28.4% compared to 30.6% in the antidepressant group. There was no significant difference between the two groups (RR = 0.84, 95% CI = 0.65-1.10, p = 0.21), as shown in Figure 3. No significant heterogeneity was reported among the study results (I^2^ = 23%, p = 0.26).

We conducted a subgroup analysis comparing the combination of ECT and antidepressant therapy with antidepressant therapy alone and ECT alone with antidepressant therapy. The subgroup analysis showed that the risk of recurrence was significantly lower in patients receiving ECT with antidepressant therapy compared to the antidepressant group (RR = 0.65, 95% CI = 0.45-0.93, p = 0.02). On the other hand, when ECT was given alone, the risk was higher in the ECT group compared to the antidepressant group, although the difference was not statistically significant (RR = 1.17, 95% CI = 0.79-1.75).

MMSE Score

Four studies compared the change in the MMSE score from baseline between two study groups at the end of trials. No significant difference was found between the two groups (MD = -1.63, 95% CI = -4.98, 1.73, p = 0.34). Significant heterogeneity was found among the study results (I^2^ = 67%, p = 0.06).

Discussion

The present meta-analysis showed that ECT, in combination with pharmacotherapy, effectively reduced the risk of relapse and recurrence at the end of the study trial. On the other hand, when ECT was given alone, it showed no significant difference between patients in the ECT group and the pharmacotherapy group in terms of reducing the risk of relapse or recurrence. Only one study in this meta-analysis assessed ECT alone to pharmacotherapy, and this study did not find any difference between the two groups. Our meta-analysis showed superior efficacy of ECT with a combination of pharmacotherapy than ECT or pharmacotherapy alone. This shows a potential synergistic role for ECT and psychotropic drugs in the maintenance treatment of depression.

The meta-analysis conducted by Elias et al. included five RCTs and reported similar findings [18]. A systematic review of randomized controlled trials by Sackeim et al. [19] found that ECT was not significantly more effective than antidepressant therapy alone in preventing depression recurrence. Similarly, a meta-analysis by Dong et al. [20] found that ECT was superior to maintenance antidepressant therapy in preventing depression recurrence. However, our meta-analysis is an updated analysis and includes recently conducted RCTs as well.

The dose and frequency of maintenance and continuation of ECT varied within the studies and from study to study. It can be difficult to gradually move patients from the acute phase of ECT to the continuation phase because they may exhibit early indicators of recurrence. The majority of the studies in this meta-analysis employed a treatment protocol that included weekly ECT for a month, followed by fortnightly ECT for a month or more [12-14,16], and in some cases, monthly ECT [17]. Most of the included studies in the present meta-analysis utilized monthly ECT. Different non-randomized and retrospective studies showed that intervals of one to two months might be optimal for maintenance ECT [18]. When it comes to electrical stimulus dosing, the dose can be established before starting c-ECT by re-titration, and the same dose can be used for as long as patients maintain clinical remission.

Cognitive functions were evaluated using the MMSE. It is a tool that does not evaluate impairment of memory, an adverse effect of significant interest with ECT [21]. The present meta-analysis found that no significant difference was found between the two study groups in terms of change in MMSE score from baseline.

International guidelines showed that maintenance and continuation treatment with ECT is considered for patients who have responded to ECT for acute depression [22]. Other guidelines based on limited evidence suggest that continuation ECT is more efficient than using antidepressants alone [11]. The results of the current study can be seen as adding to the understanding of the role of ECT in the treatment of depression, as well as providing information on the specific impact of ECT on the prevention of depression recurrence in adults with major depressive disorders. These findings can be used to inform future treatment recommendations and help healthcare providers make informed decisions about the use of ECT in the treatment of depression.

Study limitations

The limitations of this meta-analysis must also be considered when interpreting the results. First, the number of studies included in the meta-analysis was relatively small, which could affect the robustness of the results. Second, the heterogeneity among the studies in terms of the patient population, ECT technique, and study design may have influenced the results. Third, the follow-up periods of the included studies were relatively short, and the long-term effects of ECT on recurrence and MMSE scores were not considered. These limitations highlight the need for further studies with larger sample sizes and longer follow-up periods to confirm the results of this meta-analysis.

## Conclusions

The results of this meta-analysis suggest that ECT alone or in combination with an antidepressant medication does not significantly impact the incidence of recurrence in adults with major depressive disorder when compared with antidepressant medication alone. The results also suggest that there is no significant difference in change in MMSE scores between the two groups. However, the limited number of studies, heterogeneity among study populations and design, and short follow-up periods highlight the need for further research in this area. Despite these limitations, the results of this meta-analysis can inform clinical decision-making and provide valuable insights into the role of ECT in the treatment of depression. It is important to consider individual patient characteristics and the potential risks and benefits of ECT when making treatment decisions.
